# Supplementary material for: How faculty define quality, prestige, and impact of academic journals
Source: PLoS One. 2021 Oct 28;16(10):e0257340. doi: 10.1371/journal.pone.0257340 (PMC8553056; doi:10.1371/journal.pone.0257340)
Supplement: S1 Table — Overview of the participants’ definition of Quality, Prestige and Impact by their gender, institution type, and age. The color scale illustrates the distribution of responses, where green indicates a high percentage of responses and red indicates a low percentage of responses. (DOCX) [file pone.0257340.s001.docx]

**Supporting Information**

**
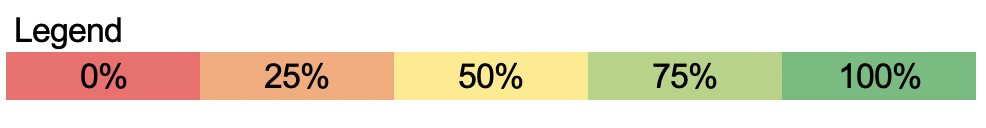
S1 Table 1.** Breakdown of definitions by demographic characteristics. *Overview of the participants’ definition of Quality, Prestige and Impact by their gender, institution type, and age.*  The color scale illustrates the distribution of responses, where green indicates a high percentage of responses and red indicates a low percentage of responses.

|  |  | Gender | |  | Institution Type | |  | Age | | | | |
| --- | --- | --- | --- | --- | --- | --- | --- | --- | --- | --- | --- | --- |
|  |  | Male | Female |  | R-type | M-type |  | 25–34 | 35–44 | 45–54 | 55–64 | 65+ |
|  | N = | 136 | 154 |  | 202 | 91 |  | 25 | 82 | 98 | 62 | 23 |
| High Quality | Impact factor and metrics | 14.7% | 8.4% |  | 13.4% | 7.7% |  | 8.0% | 9.8% | 14.3% | 4.8% | 30.4% |
|  | Quality and relevance | 34.6% | 37.0% |  | 35.1% | 35.2% |  | 36.0% | 37.8% | 32.7% | 38.7% | 34.8% |
|  | Readership | 2.9% | 1.9% |  | 3.0% | 1.1% |  | 4.0% | 1.2% | 2.0% | 4.8% | 0.0% |
|  | Reputation | 7.4% | 9.1% |  | 8.4% | 8.8% |  | 4.0% | 7.3% | 12.2% | 9.7% | 0.0% |
|  | Review process | 40.4% | 43.5% |  | 40.1% | 47.3% |  | 48.0% | 43.9% | 38.8% | 41.9% | 34.8% |
|  |  |  |  |  |  |  |  |  |  |  |  |  |
|  | N = | 125 | 129 |  | 184 | 76 |  | 20 | 77 | 85 | 55 | 22 |
| Prestige | Impact factor and metrics | 16.8% | 16.3% |  | 19.0% | 17.1% |  | 30.0% | 19.5% | 18.8% | 12.7% | 18.2% |
|  | Quality and relevance | 13.6% | 10.9% |  | 12.0% | 13.2% |  | 10.0% | 10.4% | 14.1% | 16.4% | 4.5% |
|  | Readership | 4.8% | 4.7% |  | 4.3% | 5.3% |  | 0.0% | 3.9% | 7.1% | 5.5% | 0.0% |
|  | Relation to associations | 6.4% | 2.3% |  | 4.9% | 2.6% |  | 0.0% | 1.3% | 4.7% | 5.5% | 13.6% |
|  | Reputation | 40.0% | 47.3% |  | 46.2% | 35.5% |  | 35.0% | 45.5% | 36.5% | 47.3% | 54.5% |
|  | Review process | 18.4% | 18.6% |  | 13.6% | 26.3% |  | 25.0% | 19.5% | 18.8% | 12.7% | 9.1% |
|  |  |  |  |  |  |  |  |  |  |  |  |  |
|  | N = | 119 | 121 |  | 165 | 77 |  | 18 | 75 | 74 | 55 | 22 |
| High Impact | Impact factor and metrics | 50.4% | 47.1% |  | 52.1% | 42.9% |  | 55.6% | 53.3% | 47.3% | 43.6% | 50.0% |
|  | Impact on academia | 15.1% | 17.4% |  | 15.8% | 15.6% |  | 16.7% | 10.7% | 17.6% | 20.0% | 18.2% |
|  | Impact outside academia | 10.9% | 9.9% |  | 9.1% | 14.3% |  | 5.6% | 10.7% | 8.1% | 16.4% | 9.1% |
|  | Quality | 8.4% | 7.4% |  | 9.1% | 5.2% |  | 5.6% | 5.3% | 6.8% | 12.7% | 9.1% |
|  | Readership | 15.1% | 18.2% |  | 13.9% | 22.1% |  | 16.7% | 20.0% | 20.3% | 7.3% | 13.6% |
