# Supplementary material for: How faculty define quality, prestige, and impact of academic journals
Source: PLoS One. 2021 Oct 28;16(10):e0257340. doi: 10.1371/journal.pone.0257340 (PMC8553056; doi:10.1371/journal.pone.0257340)
Supplement: S2 Table — Overview of the participants’ definition of Quality, Prestige, and Impact by discipline. The color scale illustrates the distribution of responses, where green indicates a high percentage of responses and red indicates a low percentage of responses. (DOCX) [file pone.0257340.s002.docx]

**Supplemental Information**

**S2 Table.** Breakdown of definitions by demographic characteristics. *Overview of the participants’ definition of Quality, Prestige, and Impact by discipline.* The color scale illustrates the distribution of responses, where green indicates a high percentage of responses and red indicates a low
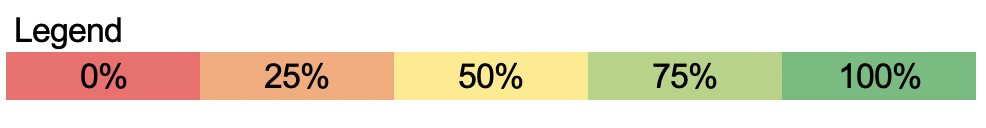
percentage of responses.

|  |  | Discipline | | | |
| --- | --- | --- | --- | --- | --- |
|  |  | Multi-disciplinary | Social Sciences and Humanities | Physical Sciences and Mathematics | Life Sciences |
|  | N = | 30 | 142 | 55 | 63 |
| High Quality | Impact factor and metrics | 16.7% | 11.3% | 7.3% | 14.3% |
|  | Quality and relevance | 40.0% | 36.6% | 32.7% | 34.9% |
|  | Readership | 0.0% | 2.1% | 3.6% | 3.2% |
|  | Reputation | 10.0% | 9.2% | 12.7% | 3.2% |
|  | Review process | 33.3% | 40.8% | 43.6% | 44.4% |
|  |  |  |  |  |  |
|  | N = | 24 | 130 | 47 | 58 |
| Prestige | Impact factor and metrics | 16.7% | 18.5% | 12.8% | 24.1% |
|  | Quality and relevance | 12.5% | 13.1% | 17.0% | 6.9% |
|  | Readership | 4.2% | 5.4% | 4.3% | 3.4% |
|  | Relation to associations | 4.2% | 3.8% | 6.4% | 3.4% |
|  | Reputation | 45.8% | 37.7% | 44.7% | 51.7% |
|  | Review process | 16.7% | 21.5% | 14.9% | 10.3% |
|  |  |  |  |  |  |
|  | N = | 22 | 125 | 45 | 52 |
| High Impact | Impact factor and metrics | 40.9% | 51.2% | 55.6% | 42.3% |
|  | Impact on academia | 27.3% | 12.8% | 22.2% | 13.5% |
|  | Impact outside academia | 18.2% | 10.4% | 6.7% | 11.5% |
|  | Quality | 4.5% | 11.2% | 0.0% | 7.7% |
|  | Readership | 9.1% | 14.4% | 15.6% | 25.0% |
