# Supplementary material for: How faculty define quality, prestige, and impact of academic journals
Source: PLoS One. 2021 Oct 28;16(10):e0257340. doi: 10.1371/journal.pone.0257340 (PMC8553056; doi:10.1371/journal.pone.0257340)
Supplement: Attachment — Submitted filename: Response to Reviewers.docx [file pone.0257340.s004.docx]

Dear Editor and Reviewers,

Thank you and the two reviewers for the helpful feedback on our manuscript “How faculty define quality, prestige, and impact in research”. We are pleased to resubmit our manuscript with the requested revisions. Below you will find the reviewers’ feedback (in gray) interspersed with a description of how we addressed each of the points raised (in black).

We would like to take this opportunity to express our appreciation to the reviewers for their thoughtful feedback. We are convinced that we have adequately addressed all the expressed concerns and that the manuscript has been improved as a result of this process.

Sincerely,

Juan Pablo Alperin

Assistant Professor, Publishing

Associate Director, Public Knowledge Project

Director, Scholarly Communications Lab

Simon Fraser University

Reviewer #1: Review of PONE-D-21-10048

This manuscript reports on the answers to an open survey question about how faculty of universities in the USA and Canada would define quality, prestige and impact of academic journals. The article is interesting and well-written but a number of major and minor concerns should be adequately responded to with a view to optimize clarity and relevance of the manuscript.
Major concerns
♣ It’s not very clear what quality, prestige and impact refer to: research (as in the title), journals (as asked in the survey), to the publication oeuvre of an individual researcher (as one would expect given the focus on review, promotion, and tenure (RPT) processes), or to all of these categories combined (which would be strange and confusing). Please clarify and apply the choice consistently throughout the manuscript.

We strongly agree with this comment and have now changed the title and made several edits to make it clear that the focus of the paper are the definitions of quality, impact, and prestige of academic journals, as was asked in the survey. Edits include the use of “academic journals” in the title, the abstract, on p. 3 (lines 16-19; line 20), p. 7 (line 10), p. 9 (line 5), p. 12 (line 17), and page 13 (line 10). We are certain that these small additions, when combined with the new title and already present instances of the phrase “of academic journals,” make amply clear the object study.

♣I agree that having a clear definition or description of core concepts used in the assessment of research and researchers is a necessary starting point but please also discuss the following.
o The meta-question whether quality, prestige and impact are the main concepts we need to look at. You allude to this a bit in the Introduction section but remain silent in the Discussion section and the Abstract.
o The practical question how quality, prestige and impact can be operationalized. This seems to be the main focus of your respondents (and less so the conceptual definitions).

We acknowledge these comments and feel that they are already adequately addressed in the manuscript. In terms of justification for the use of these terms, we note in the introduction that the three terms/concepts are important ones in research assessment, citing a comprehensive literature review from our team (Schimanski & Alperin, 2018). Subsequently, we present the statistics of how prevalent the terms are in RPT guidelines (p. 3; lines 26-31). The presence of the terms in the literature and in the documents seem sufficient to warrant the closer examination of these terms, not necessarily as the “main concepts”, but as important ones.

With regards to the practical question of how these concepts are to be operationalized, we do not think it is our place to delve into the matter. It is true that many respondents focused on operational definitions, but we note that not all of them do. For example, defining “High Quality” or “Prestige” by “Reputation” is using one concept to define another. We do discuss these circular definitions, as well as the focus on a citation-centric operationalization of the concepts, to a great extent in the discussion for this reason.

We do not disagree with the reviewers on the points raised, and hope this response is sufficient to explain how we think the points are adequately addressed in the manuscript.

♣ Maybe also mention (in the Discussion section) the best practices presented by DORA on their website and the recently introduced Hong Kong principles:
Moher D, Bouter L, Kleinert S, Glasziou P, Sham MH, Barbour V, Coriat AM, Foeger N, Dirnagl U. The Hong Kong principles for assessing researchers: fostering research integrity. PLoS Biology 2020; 18: e3000737. (<https://journals.plos.org/plosbiology/article?id=10.1371/journal.pbio.3000737>) (translated in Chinese, German, Portuguese)

Great suggestion. We have added several lines to this effect, including the mention of the Hong Kong Principles. In addition to mentioning the initiatives (p. 14; lines 25-29), we have added the following (pp. 15; lines 16-29).

DORA advises against using any journal-level metrics, and the JIF in particular, as measures of research quality in evaluations. It recommends instead relying on a variety of article-level metrics and qualitative indicators to better assess individual works. The Leiden Manifesto cautions against over-reliance on and incorrect uses of quantitative metrics, and emphasizes the importance of qualitative assessments as well as contextual and disciplinary considerations. HuMetricsSS encourages the development of value-based assessments and proposes a definition of ‘quality’ as “a value that demonstrates one’s originality, willingness to push boundaries, methodological soundness, and the advancement of knowledge” and the Hong Kong Principles recommend assessing research practices, such as complete reporting or the practices of open science, instead of citation-based metrics.

♣ I’m frankly puzzled that both the documents you analysed earlier and the faculty you surveyed don’t focus much more on the H-index. Although this measure – which is frequently used in researcher assessments in Europe – is also deeply flawed it’s clearly superior to the JIF for assessing researchers for two reasons: 1) the H-index concerns the whole publication oeuvre of an individual, and 2) the H-index is based on the actual citations to the articles the researcher at issue published. Please comment on this in the Discussion section.

The term `H-index` is included in the concept “metrics” that we searched for in the RPT documents, but it was, in fact, very seldomly used. This was not surprising to us, perhaps because the h-index is not very widely adopted in the North American context, but also because it does not apply to academic journals (despite Google Scholar’s use of it in this way). Now that the paper makes it much clearer that the concepts studied refer to academic journals and not the oeuvre of a researcher, we don’t think it would be appropriate to dwell on it in the paper. Data about the number of mentions of the h-index are available in our Open Data set that accompanies this series of papers, starting with Alperin et al., 2018.

♣ It’s not so clear to me why you would expect differences between subgroups defined by demographic characteristic and why that would be interesting or relevant to know.

There is empirical evidence that publication decisions and practices are affected by demographic characteristics. We have now added a few lines in the literature review (pp. 4-5) that read as follows:

Further complicating our understanding of faculty’s understanding and use of these concepts is the role that demographic characteristics—such as gender and career stage—significantly impact how scholarship is perceived and practiced (e.g., Hammarfelt, 2017; Holman et al., 2018). In our own previous work (Niles et al., 2020), for example, we found that women are more likely to publish fewer articles than men but often assign more importance to the number of publications than their male peers.

♣ It’s also not clear why you would expect a high correlation between the views of faculty and the policy documents in their university. On a slightly cynical note: who is reading these documents? My guess is hardly anyone.

In the North American context, these documents are highly scrutinized by the junior faculty (i.e., Assistant Professors) who are going up for promotion and tenure. One of our study’s main contribution is to provide evidence that, despite this nominally important role, the faculty serving on these committees might not be reading these documents, or, if they are, the documents are not shaping their understanding of the terms.

♣ The manuscript is much longer than necessary and should be shortened substantially by e.g.
o Removing lines 16-30 on page 5 and lines 1-21 on page 6. This lengthy description of earlier work doesn’t belong in the Methods section (or elsewhere in the article) and can be replaced by one or two sentences that only explain what’s really of direct importance to understand the methods of what is reported in this manuscript.
o Figures 1-3 have little informative value and can easily be removed when the percentages reported are transferred to tables 1-3.
o Table 4 and 5 can better be moved to the digital supplements as their message (‘no significant differences between subgroups’) is already well described in two lines in the text.

We are more than happy to reduce the replication of methods that are already published elsewhere, and have cut several lines from the beginning of the methods, leaving only the details on the survey, which we feel are most relevant. We also agree with the removal of Figures 1-3, and have, instead, added a columns to Tables 1-3, as suggested. Thank you.

We would be willing to move Tables 4-5 to supplementary material, but do feel like they best belong in the manuscript. While there are no significant statistical differences, we find the data a valuable contribution and, since the lack of difference goes contrary to expectations, we think it is a significant-enough result to warrant being in the main manuscript.

Minor concerns
♣ What was the response of the survey? It’s unclear how many invitations were sent. And how many participants answered the open question on which this manuscript is based? Respondents often skip open questions. Please produce a flow chart.

In simplifying the methods, we have now added details on the number of invitations and the response rates, to both the survey as a whole and to this set of questions in particular. We opted to add this in text (as opposed to a flow chart) since the information is fairly straight-forward. It now reads as follows:

To conduct this study, we sent an online survey to 1,644 faculty members from 334 academic units from 60 universities from Canada and the United States. As described in greater detail in Niles et al. (2020), we created this contact list based on a random sample of universities from which we have previously collected RPT guidelines.

A few lines further down, it reads:

Of the 338 responses, 249 (74%) responded to this set of open-ended questions, for an effective response rate of 15% of the 1,644 invitations that were sent.

♣ Was your survey pre-registered? Please clarify and add the pertaining link if you did.

It was not pre-registered.

♣ Please keep the order of quality, prestige and impact uniform throughout the text and also order tables 1, 2 and 3 likewise.

We have now done so throughout the manuscript.

♣ In table 1 you say ‘definitions of High Quality’ in the title but talk about ‘definitions of High Impact in the note directly below the table. That last formulation seems to be wrong.

This has now been corrected.

♣ It’s not clear what the different colours in table 4 and 5 indicate.

We have now added the following text to the table captions, as well as the following legend below the tables (perhaps only one of these two is necessary).

The color scale illustrates the distribution of responses, where green indicates a high percentage of responses and red indicates a low percentage of responses.

I sign my reviews:
Lex Bouter, Amsterdam University Medical Centers and Vrije Universiteit Amsterdam

Reviewer #2: The research shows that absolute general definitions of quality, impact and prestige which may be used to compare researchers and research within disciplines and cross academia may be difficult if not impossible to define. It is also because of this that in academia we started to use 'objective' indicators and metrics which have been shown however to be poor proxies for quality and excellence. In the attempt to go beyond these flawed metrics there are now many initiatives to design meaningful ways to evaluate research. Based on their observation in this paper, the authors feel that is problematic since quality, excellence and prestige are ill-defined and researchers have many different views of it and most times rely on the flawed classical metrics, i.e. papers and where they are published and cited.

I would suggest to the authors to include a discussion of those initiatives that really are trying to deal with this problem. In the introduction they cite several of the papers that discuss this, f.i . Aksnes et al 2019, Hicks et al 2015 which is The leiden manifesto and Moher et al 2018.

Thank you for this suggestion. We have now done so in the discussion, as discussed above (particularly on p. 14; lines 16-29)

Instead of looking for the impossible, i.e. absolute, timeless measures for quality and prestige, these initiatives start with the conclusion that quality, excellence and prestige are context dependent. Research quality, its products and evaluation are highly context dependent which makes direct comparisons of historians, philosophers and chemists and even researchers within for instance the domain of biomedical and health research, irrelevant. It is about 'rigor, plausibility, originality, societal value' (Asknes 2019) but in a given research setting, with a strategy, aim, specific goals, a process (f. i .Open Science practices) and if applicable actions in the corresponding societal context (Nowotny et al, Rethinking Science, 2001).

To say that research is Excellent or Good requires from peers/reviewers, a narrative, a motivation of judgement of strengths and weakenesses based on reading and understanding of the content of the work.

This is the approach taken in the recently adopted National Strategic Evaluation Protocol (SEP) in The Netherlands
https: //[www.vsnu.nl/files/documenten/Domeinen/Onderzoek/SEP_2021-2027.pdf](http://www.vsnu.nl/files/documenten/Domeinen/Onderzoek/SEP_2021-2027.pdf)

We very much agree with this reviewer’s view and believe that the research presented here (alongside the other publications from this project) help to confirm this perspective by showing, through concrete empirical evidence, some of the pernicious effects of using seemingly objective measures and standard definitions for evaluations that should be context specific.
