# Supplementary material for: How faculty define quality, prestige, and impact of academic journals
Source: PLoS One. 2021 Oct 28;16(10):e0257340. doi: 10.1371/journal.pone.0257340 (PMC8553056; doi:10.1371/journal.pone.0257340)
Supplement: Attachment — Submitted filename: Response to Reviewers R&R2.docx [file pone.0257340.s005.docx]

Dear Editor and Reviewers,

Thank you and the two reviewers for this rapid second review. We apologize for our own delay in returning our manuscript “How faculty define quality, prestige, and impact in research”. The response came while many of us were away on vacation and it is only now that we were all able to review our response.

We are pleased to resubmit our manuscript with the requested revisions. As you can see in the attached manuscript, we have moved the two tables to supplementary materials and made substantial additions to directly address the remaining reviewer comment in the discussion. You will now find additions that directly include the reviewer’s suggestions on p. 13 (lines 15-16; 19-29), p. 14 (lines 13-17; 22-28), as well as in a line in the abstract.

We are certain these additions would be met with agreement by the reviewer, as they substantially incorporate their views into the discussion and conclusions of our work. We trust you will agree.

Sincerely,

Juan Pablo Alperin

Associate Professor, Publishing

Associate Director, Public Knowledge Project

Director, Scholarly Communications Lab

Simon Fraser University
